# Altered GNAS imprinting due to folic acid deficiency contributes to poor embryo development and may lead to neural tube defects

**DOI:** 10.18632/oncotarget.22731

**Published:** 2017-11-28

**Authors:** Li Wang, Shaoyan Chang, Zhen Wang, Shan Wang, Junsheng Huo, Gangqiang Ding, Rui Li, Chi Liu, Shaofang Shangguan, Xiaolin Lu, Ting Zhang, Zhiyong Qiu, Jianxin Wu

**Affiliations:** ^1^ Beijing Municipal Key Laboratory of Child Development and Nutriomics, Capital Institute of Pediatrics, Beijing, P.R. China; ^2^ National Institute for Nutrition and Health, Chinese Center for Disease Control and Prevention, Beijing, P.R. China

**Keywords:** GNAS imprinting gene, folic acid, embryo development, neural tube defects, methylation

## Abstract

Disturbed epigenetic modifications have been linked to the pathogenesis of Neural Tube Defects (NTDs) in those with folate deficiency during pregnancy. However, evidence is lacking to delineate the critical region in epigenome regulated by parental folic acid and mechanisms by which folate deficiency affects normal embryogenesis. Our data from clinical samples revealed the presence of aberrant DNA methylation in GNAS imprinting cluster in NTD samples with low folate concentrations. Results from mouse models indicated that the establishment of GNAS imprinting was influenced by both maternal and paternal folate-deficient diets. Such aberrant GNAS imprinting was present prior to the gametogenesis period. Imprinting in Exon1A/GNAS gDMR was abolished in both spermatozoa and oocytes upon treating with a parental folate-deficient diet (3.6% in spermatozoa, 9.8% in oocytes). Interestingly, loss of imprinting in the GNAS gene cluster altered chromatin structure to an overwhelmingly open structure (58.48% in the folate-free medium group vs. 39.51% in the folate-normal medium group; *P* < 0.05), and led to a disturbed expression of genes in this region. Furthermore, an elevated cyclic AMP levels was observed in folate acid deficiency group. Our results imply that GNAS imprinting plays major roles in folic acid metabolism regulation during embryogenesis. Aberrant GNAS imprinting is an attribute to NTDs, providing a new perspective for explaining the molecular mechanisms by which folate supplementation in human pregnancy provides protection from NTDs.

## INTRODUCTION

There is an emerging concept that early life environment may have widespread consequences on individual’s health later in life, starting from zygote stage, through infancy, to adulthood [1]. Epidemiological and animal studies have shown that there is a close link between birth defects and exposure to environmental factors, including diet, deficiency in nutrition, and toxic chemical [2–4]. Among these, considerable attention has been paid to folic acid because of its key role in maintaining healthy embryo development [5, 6]. Folic acid deficiency has long been recognized as an important contributor to developmental anomalies [4, 7–10], and has been correlated with increased risk of intrauterine growth restriction (IUGR) and neural tube defects (NTDs), as well as cardiac and limb abnormalities. Our previously work also indicated that lower concentrations of maternal folate are related to an increased risk of NTDs [11]. Folate intake and availability play a crucial role in epigenetic programming through the 1-C metabolic pathway, and the epigenetic regulation involved has been regarded a fundamental aspect in studying early development and disease. Maternal folate deficiency during pregnancy has been implicated in adverse pregnancy outcomes due to the induction of DNA hypomethylation, and subsequently an altered expression of apolipoprotein AI and interferon-gamma in the spleen and placenta [10, 12–14]. There is growing evidence that paternal folic acid levels can also affect the epigenetic status of the genome [15–17]. Paternal epigenetic alterations can lead to lethal mutations [18] and also result in poor embryonic development [19–21]. Parental epigenetic profile established during environmental exposures will be passed on by the gametes, ultimately affecting the development of offspring [21]. Although the effects of folate deficiency on embryo development are extensive, profound, and pleiotropic, evidence is lacking to delineate the critical region in epigenome regulated by parental folic acid and mechanisms by which folate deficiency affects normal embryogenesis.

There exist critical windows in development when the epigenome is more susceptible to the exposure to environmental factors for the induction of epimutations [22]. However, only portion of the established parental epigenetic profile is transferrable from parents to their offspring, known as imprinted genes. Imprinted genes form one class of elements within the human genome whose methylation patterns are sensitive to malnutrition in early embryonic development [23]. Imprinting marks are established in the germline of the parents and are maintained through mitotic cell divisions in the somatic cells of an organism [24]. Imprinted genes are sensitive to parental nutrition and are potentially evolved in the balance of parental resource allocation to the offspring [25]. Thus, appropriate imprinting of certain genes is important for normal development [26–28].

One of the most important regions for fetal development is the GNAS imprinting cluster, which is well conserved between the human and mouse. Three differentially methylated regions (DMRs) have been found in the GNAS imprinting cluster: an extensive germline maternally methylated region at the Nespas promoter (Nespas gDMR); a germline maternally methylated region at the Exon1A promoter (Exon1A/GNAS gDMR); and a paternally methylated region spanning the Nesp promoter (Nesp DMR) [29, 30]. GNAS encodes the alpha subunit of a major heterotrimeric G-signaling protein, which is active in fetal growth and development [31, 32]. GNAS knockout mice show an imprinting defects phenotype, with an increased insulin sensitivity and adipocyte hypertrophy in the null animals [26]. Data from studies on placentas gene expression in samples from pregnancies with IUGR have demonstrated down regulation of GNAS [33–35], however, there is no prior studies conducted to elucidate whether the GNAS imprinting cluster plays a role in periconceptional folate deficiency-related IUGR.

In this study, the effect of folic acid deficiency on GNAS imprinting and developmental diseases was assessed in human NTD samples with lower folate concentrations. Furthermore, we investigated the effect of parental dietary folate deficiency on offspring development and GNAS imprinting regulation using a mouse model to validate the translational value of the observation from case studies. The influences of maternal and paternal dietary folate deficiency were evaluated separately to delineate their respective effect on gDMR methylation modifications. Subsequently, levels of gene expression controlled by imprinting were analyzed, and signaling pathways downstream of GNAS imprinting gene were evaluated to assess their effects on development.

## RESULTS

### Loss of imprinting in GNAS gene cluster in human fetal NTD samples with lower folate concentrations

It has been hypothesized that lower levels of folic acid during pregnancy may lead to abnormal gene imprinting which is involved in pathology of human NTDs. As the first step to elucidate the cause-effect relationship of these three, we evaluated the GNAS imprinting status in six NTD fetal brain samples with low brain folate concentrations and six control samples with higher brain folate concentrations. All selected NTDs samples were diagnosed with multi-system dysontogenesis, including aberrations to the urinary system, circulatory system or talipesequinovarus. Detailed pathological manifestations in these samples are listed in Table 1. Control samples were collected from fetuses aborted for nonmedical reasons, devoid of any malformations.

**Table 1:** Folate concentrations and methylation levels of the DMRs in the GNAS imprinted gene cluster of human fetal brains, comparing NTD and control samples

Sample	Pathological diagnosis	Folate con. (ng/mg)	Methylation level (%)		
			Nespas DMR	Exon1A/GNAS DMR	Nesp DMR
NTD 1	Bifid spine; Renal fusion; TalipesEquinovarus; Hydrocephaly	0.02	38.58	24.08	44.80
NTD 2	Bifid spine; Hydrocephaly; TalipesEquinovarus	0.03	25.17	18.00	37.00
NTD 3	Anencephalus; Occipital meningealencephalocele; IUGR	0.03	36.95	42.17	39.13
NTD 4	Bifid spine; Unilateral agenesis of kidney and ureter; TalipesEquinovarus	0.04	39.11	/	43.60
NTD 5	Occipital meningeal encephalocele	0.04	34.83	43.08	37.93
NTD 6	Bifid spine; Hydrocephaly; Congenital heart disease	0.04	33.11	45.67	36.93
Control 1	/	0.23	36.72	39.25	43.21
Control 2	/	0.24	37.89	40.33	32.73
Control 3	/	0.24	40.61	46.67	33.78
Control 4	/	0.25	40.94	38.50	35.13
Control 5	/	0.26	44.89	36.08	37.27
Control 6	/	0.47	42.13	/	38.17
Aver NTD^1^		0.03 ± 0.01^*^	34.95 ± 4.79^*^	39.67 ± 4.01	39.90 ± 3.45
Aver Con^1^		0.28 ± 0.09	40.53 ± 2.94	39.70 ± 2.25	36.72 ± 3.79
*p*		0.00	0.03	0.99	0.16

^1^All values are the mean ± SD.

^*^Significantly different between NTDs and controls, *P* ≤ 0.05 by Student’s *t*-test.

**Figure 1:**
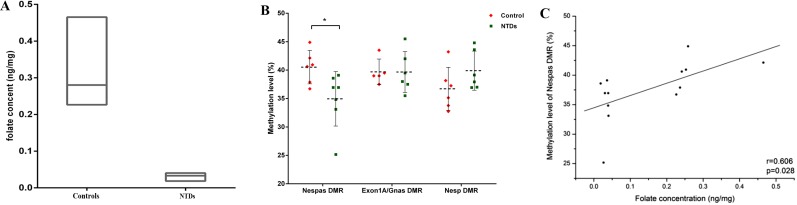
Loss of imprinting in the GNAS gene cluster in human fetuses with neural tube defects (NTDs) was associated with lower folate concentrations (**A**) Folate concentrations in NTDs and controls. (**B**) comparison of methylation levels of three DMRs in the GNAS gene cluster between NTDs and controls, ^*^P < 0.05. (**C**) correlation between folic acid and methylation levels of the Nespas DMR in human fetal brains.

Folic acid concentrations and GNAS methylation levels in fetal brain tissues were compared between the two groups. Significantly lower levels of folate were observed in the NTD samples (0.03 ± 0.01 ng/mg) compared to that in the controls (0.28 ± 0.09 ng/mg) (*P <* 0.05). In addition, there was a significant reduction in methylation level, 34.95 ± 4.79% in NTDs vs. 40.53 ± 2.94% in controls (*P <* 0.05), in the Nespas DMR, one of the three DMRs located in the GNAS imprinting cluster (Figure 1). Within the Nespas DMR, all 27 CpG sites except for the 3rd and 11th, had a downward trend in methylation levels in the NTD samples compared to controls (Supplementary Figure 1). Furthermore, a significantly positive correlation was noted between methylation level of the Nespas DMR and folic acid concentration (Figure 1, *P =* 0.028). However, no statistically significant difference in methylation levels were observed in EXON1A/GNAS gDMR or the NESP DMR, between samples from NTDs and controls (Figure 1).

### Disturbed methylation modifications in the GNAS imprinting cluster in mouse offspring under folic acid deficiency conditions

**Figure 2:**
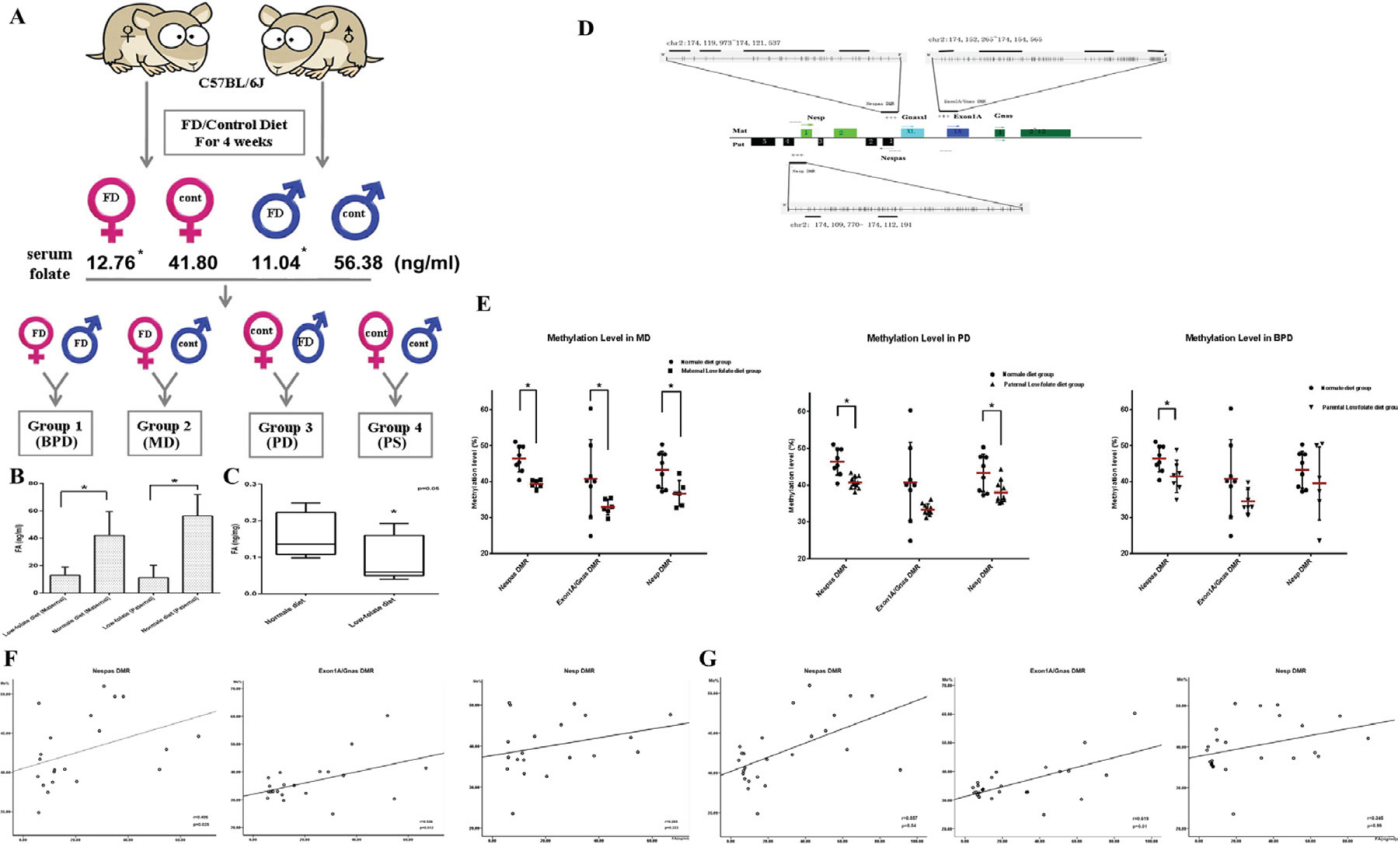
Folic acid deficiency in pregnancies with disturbed imprinting modifications in the GNAS gene cluster in offspring (**A**) Flowchart of folate-deficient mice treated and mated to each other in four different groups: paternal folate-supplemented × maternal folate-supplemented (PS) group; paternal folate-deficient × maternal folate-supplemented (PD) group; paternal folate-supplemented × maternal folate-deficient (MD) group; and paternal folate-deficient × maternal folate-deficient (BPD) group. (**B**, **C**) Folic acid concentrations in parental peripheral blood and placenta compared between folate-deficient groups and controls. ^*^P < 0.05. (**D**) Schematic diagram of the GNAS imprinted gene cluster. The maternal (Mat) and paternal (Pat) alleles of GNAS are depicted with alternative first exons NESP (Nesp), XL (GNASxl), 1A, and GNAS splicing to common exons 2–12. The rectangles represent exons in the GNAS cluster and the arrows show the transcription start sites of the different genes. The + and – symbols represent gain or loss of methylation of the DMRs, respectively. Each vertical line represents an individual CpG site. The locations of the amplicons used for methylation analysis are shown by the short bars under each DMR. (**E**) Methylation levels of three DMRs of GNAS imprinting cluster between MD, PD, BPD and controls. ^*^P < 0.05. (**F**) Association between the methylation level of three GNAS DMRs and folic acid concentrations in maternal peripheral blood. (**G**) Association between methylation levels of the three GNAS DMRs and folic acid in paternal peripheral blood.

To further investigate the potential effects and mechanism of action for folic acid in parents and fetuses, we utilized folate-deficient mouse models. As shown in Figure 2A and illustrated in Materials and Methods, four different groups of mating pairs were used, and folic acid concentrations in parental peripheral blood and placentas in mice from each mating group were measured (Figure 2B, 2C). Mice fed with the FD diet had significantly lower folate concentrations in both maternal and paternal peripheral blood, compared with controls (12.76 ± 6.13 ng/ml in FD diet group vs. 41.80 ± 17.63 ng/ml in the controls in maternal blood, 11.04 ± 9.24 ng/ml in FD diet group vs. 56.38 ± 15.54 ng/ml in the control in paternal blood, respectively, *P <* 0.05). In the placentas, although the difference in folate concentration between FD diet group and controls was statically insignificant, 0.09 ± 0.06 ng/mg in FD diet group vs. 0.16 ± 0.06 ng/mg in the controls, a downward trend in folate concentration was detected in the placentas in the FD diet groups (Figure 2C).

The potential effect of a parental FD diet on pregnancy outcomes and fetal development was evaluated in each mating group. Folate deficiency during embryogenesis impaired embryonic growth, as listed in Table 2. While there were no significant differences in placental weights among the four groups, maternal and paternal FD diet appeared to each led to different developmental consequences. In the MD diet group, the rate of fetal resorption increased, consistent with previous observations that higher folate was associated with reduced risk of abortion. In the FD diet group, the crown–rump lengths of the fetuses decreased while in the BPD groups, there was a reduction of the weights of fetuses (Table 2). Our data suggest that normal folate intake in both maternal and paternal is important for embryogenesis.

**Table 2:** Status of mouse embryo development at E18.5 infolate-deficient or normal diet groups^*^

Group (*n*)	No. of Fetus	Ratio of absorbed fetus (%)	Crown-rump Length (cm)	Weight of Fetus (g)	Weight of Placenta (g)
BPD (17)	8.10 ± 2.78	8.16 ± 13.21	2.35 ± 0.13	1.17 ± 0.71^*^	0.17 ± 0.02
MD (9)	6.00 ± 3.12	22.00 ± 34.52^*^	2.25 ± 0.11	1.30 ± 0.10	0.12 ± 0.02
PD (16)	7.06 ± 2.26	4.42 ± 8.60	2.07 ± 0.10^*^	1.21 ± 0.11	0.11 ± 0.03
PS (13)	7.17 ± 2.44	3.16 ± 6.02	2.37 ± 0.22	1.24 ± 0.12	0.10 ± 0.02

^*^All values are the mean ± SD. Differences between the folate-deficient group and the control group were analyzed by Student’s *t*-test, ^*^P ≤ 0.05.

BPD: paternal folate-deficient X maternal folate-deficient groups.

MD: paternal folate-supplemented X maternal folate-deficient groups.

PD: paternal folate-deficient X maternal folate-supplementedgroups.

PS: paternal folate-supplemented X maternal folate-supplementedgroups.

To evaluate the effects of different parental folic acid deficiency on GNAS imprinting of offspring, we measured methylation levels of the three DMRs of the GNAS imprinting cluster in fetuses from all four mating groups described above. Figure 2D shows the specific regions analyzed and amplified fragments. The methylation levels of three DMRs among the four groups (MD, PD, BPD and PS) were compared separately, and the results are shown in Figure 2E and Table 3. In the MD group, all three DMRs lost their imprinting. Compared to controls, methylation levels decreased by 7.1% in Nespas DMR, 7.8% in Exon1A/GNAS DMR, and 6.7% in Nesp DMR, respectively (Figure 2E). By contrast, not all DMRs lost their imprinting in the PD and BPD groups. In the PD group, the methylation levels changed from 46.4 ± 3.7% (control) to 40.7 ± 1.7% (PD group) in the Nespas DMR, and from 43.3 ± 5.1% (control) to 38.0 ± 3.1% (PD group) in the Nesp DMR, respectively. In the BPD group, change in methylation status was only seen in the Nespas DMR, decreased from 46.4 ± 3.7% (control) to 41.4 ± 4.5% (BPD group). Overall, maternal folate deficiency seemed to have a more profound effect on methylation modification in the GNAS imprinting cluster compared to other two treated groups. In addition, there was a positive association between methylation levels in the Nespas and Exon1A/GNAS DMRs, and folic acid levels in both maternal and paternal peripheral blood (Figure 2F, 2G), further confirming the effect of folic acid levels on GNAS imprinting modifications.

**Table 3:** Methylation levels of DMRs in the GNAS imprinted gene cluster among folate-deficient and control groups

	Nespas DMR (%)	Exon1A/GNAS DMR (%)	Nesp DMR (%)
Normal diet group	46.35 ± 3.70	40.70 ± 10.93	43.25 ± 5.10
Maternal Low-folate diet group	39.32 ± 11.40^*^	32.87 ± 2.14^*^	36.64 ± 3.54^*^
Paternal Low-folate diet group	40.69 ± 1.72^*^	33.37 ± 1.52	37.97 ± 3.15^*^
Parental Low-folate diet group	41.43 ± 4.47^*^	34.50 ± 3.23	39.46 ± 10.19

All values are the mean ± SD. Differences between the folate-deficient and the normal group were analyzed by Student’s *t*-test, ^*^P ≤ 0.05.

### Folic acid deficiency disturbed GNAS imprinting starting from gametogenesis and continued through

**Figure 3:**
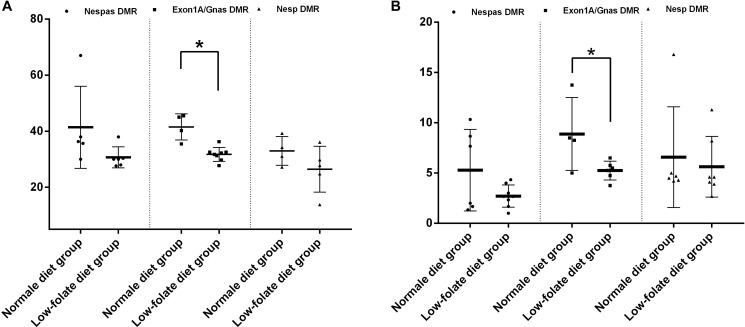
Folic acid deficiency disturbed GNAS imprinting, starting from gametogenesis in mice (**A**) Imprinting modifications of three DMRs in eggs between folic acid deficiency group and normal group. ^*^P < 0.05. (**B**) Imprinting modifications of three DMRs in sperms between folic acid deficiency group and normal group. ^*^P < 0.05.

It is commonly accepted that gene imprinting is established during gametogenesis. In this study, we further investigated the course of GNAS imprinting modifications in oocytes and spermatozoa from folic acid-deficient mice by analyzing the time course for the loss of imprinting. As shown in Figure 3, imprinting in GNAS DMRs was established in oocytes from the group fed with normal diet, with methylation levels of 35.00 ± 1.74% (Nespas DMR), 41.56 ± 2.34% (Exon1A/GNAS DMR), and 32.98 ± 2.56% (Nesp DMR), respectively (Figure 3A). A hypomethylation status of spermatozoa from the same group was maintained with less than 10% in all three DMRs (Figure 3B). As expected, in the FD groups, both oocytes and spermatozoa lost imprinting in the Exon1A/GNAS DMR, a germline DMR (31.75 ± 0.87% in FD group vs. 41.56 ± 2.34% in control for oocytes, *P* < 0.01; 5.25 ± 0.38% in FD group vs. 8.88 ± 1.81% in controls for spermatozoa, *P =* 0.04). The second germline DMR, the Nespas DMR, also showed a trend towards loss of imprinting during gametogenesis in both oocytes and spermatozoa (30.67 ± 1.54% in FD group vs. 35.00 ± 1.74% in controls for oocytes, *P* = 0.08; 2.71 ± 0.39% in FD groups vs. 5.28 ± 1.65%, in controls for spermatozoa, *P =* 0.08) (Figure 3). No significant changes were detected in Nesp DMR (Figure 3A, 3B), for which imprinting was established during embryogenesis.

Next, we evaluated GNAS imprinting status during different developmental periods. For Nesp DMR, which takes part in the second methylation wave during embryogenesis, a drifting in methylation levels was observed between development stages at oocytes, E13.5, and E 18.5 in controls (Table 4). However, little change was observed in Nesp DMR methylation levels during these three time points in MD group, the consequence of which may need further evaluation, suggesting that maternal low folate diet may block the normal establishment of normal Nesp DMR methylation during embryogenesis.

**Table 4:** Comparison loss of imprinting of GNAS gene cluster at different periods during development between the maternal low-folate diet (MD) group and controls

		Methylation level (%)		
		Nespas DMR	Exon1A/GNAS gDMR	Nesp DMR
Oocytes	Controls	35.17 ± 3.16	41.56 ± 4.68	33.00 ± 5.11
	MD	30.67 ± 3.77	31.75 ± 2.45^*^	26.50 ± 8.20
Fetal brain (E13.5)	Controls	33.50 ± 8.26	40.00 ± 4.57	40.74 ± 4.04
	MD	30.33 ± 9.25	35.50 ± 2.88^*^	39.23 ± 2.41
Fetal brain (E18.5)	Controls	38.17 ± 5.64	39.97 ± 4.95	39.63 ± 6.61
	MD	30.78 ± 6.71	31.75 ± 2.56^*^	32.42 ± 3.68^*^

No significant differences were observed in Nespas DMR at oocytes, E13.5, and E 18.5, although a similar decreased trending existed in all three phases (Table 4). Interestingly, established imprinting of the Exon1A/GNAS DMR was seen as early as during oogenesis with a methylation level of 41.56 ± 4.68%. During embryogenesis, the methylation level in fetal brain tissues remained nearly unchanged at about 40% at E13.5 and E18.5 (Table 4). However, imprinting in Exon1A/GNAS gDMR was disrupted in mice fed with maternal low-folate diet, in which methylation level decreased from 41.56 ± 4.68% in the control group to 31.75 ± 3.16% in the treated group. This decreased level remained throughout embryogenesis in brain tissues, as shown at the E13.5 and E18.5 stages in Table 4.

### Aberrant GNAS imprinting and chromatin accessibility in ESC cultured in folate-free medium

**Figure 4:**
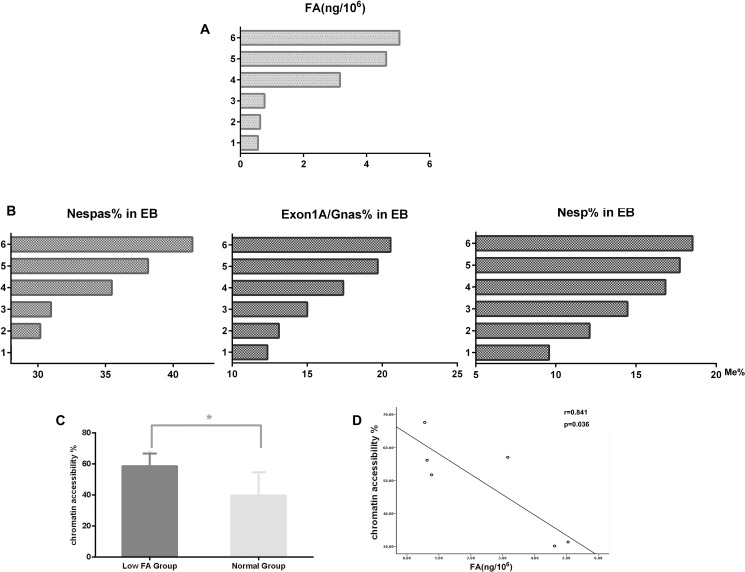
Methylation and chromatin structural alternations in EB cells with folic acid deficiency 1–3, FF medium group; 4–6, FN medium group. (**A**) Intracellular folic acid concentrations. (**B**) Methylation levels of three DMRs in EB cells with FF or FN treatment. (**C**) Alteration of chromatin accessibility between the FF medium and FN medium groups. (**D**) Correlations between chromatin accessibility and intracellular folic acid levels.

To further evaluate the effect of folic acid on GNAS imprinting, mouse ESCs were first cultured in folate-free (FF) or folate normal (FN) medium and then induced to differentiate into embryonic bodys (EBs). After 4 day incubation, a substantial reduction in methylation levels of all three DMRs within the GNAS imprinting cluster was observed when EBs were cultured in FF medium (Table 5, Figure 4A, 4B; *P* < 0.05). Furthermore, methylation alternations in all three DMRs were significantly correlated with measured folate concentrations (*r*_Nespas_= 0.979, *P =* 0.004; *r*_Exon1A/GNAS_ = 0.974, *P =* 0.001; *r*_Nesp_= 0.824, *P =* 0.044).

**Table 5:** Mean methylation levels of DMRs in the GNAS imprinted gene cluster in EBs from the folate-free (FF) medium and folate-normal (FN) medium groups

	FA conc. (ng/10^6^)	Nespas DMR (%)	Exon1A/GNAS DMR (%)	Nesp DMR (%)
FF	0.73 ± 0.18^*^	30.57 ± 0.56^*^	13.49 ± 1.36^*^	12.05 ± 2.44^*^
FN	4.09 ± 0.77	38.35 ± 3.00	19.22 ± 1.63	17.70 ± 0.84

All values are the mean ± SD. ^*^P ≤ 0.05 by Student’s *t*-test.

Results from previous studies suggest that alternation in chromatin structure surrounding specific genes might be responsible for gene silencing caused by hypomethylation. To test this hypothesis, assays on chromatin structure were conducted to compare differences in chromatin accessibility within GNAS imprinting cluster in EBs between the FF and FN groups. As shown in Figure 4C, an increased chromatin accessibility accompanied with a decreased methylation level of Exon1A/GNAS was evident in the FF group compared with the FN group (58.48% in FF vs, 39.51% in FN; *P* < 0.05). Chromatin accessibility was negatively correlated with folic acid levels in EBs (Figure 4D, *r* = 0.841; *P =* 0.036).

Taken together, by using ESCs cultured in FF medium, we demonstrated that folate deficiency could lead to aberrant GNAS imprinting and subsequently affect gene function through an altered chromatin structure.

### Increased gene expression and cyclic AMP levels with hypomethylation of the GNAS imprinting cluster

**Figure 5:**
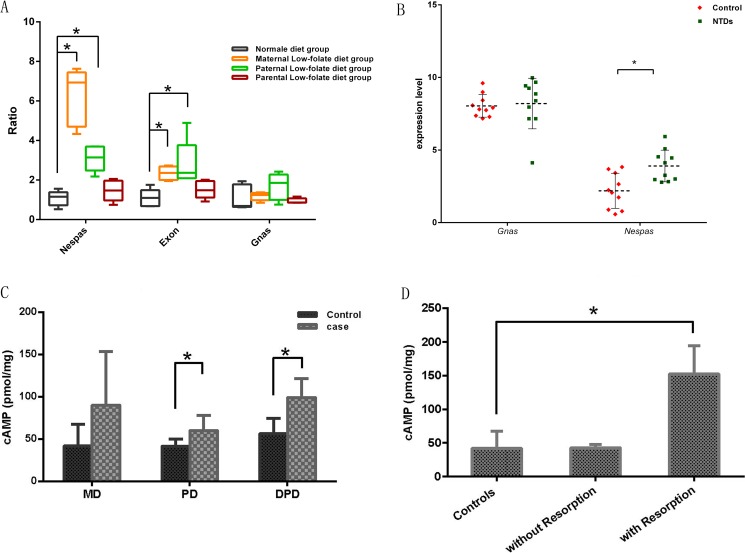
Expression level of genes controlled by the hypomethylated maternal gDMRs in mouse fetal brains (**A**) The expression levels of Nespas, Exon1A and GNAS in fetal brains. The values are means ± SDs. ^*^P < 0.05 by Student’s *t*-test. (**B**) The expression levels of Nespas and GNAS in human NTDs brain tissues. The values are means ± SDs. ^*^P < 0.05 by Student’s *t*-test. (**C**) cAMP levels in fetal brains with folic acid deficiency. ^*^P < 0.05. (**D**) cAMP levels in fetal brains with folic acid deficiency in the MD group with/without embryo resorption. ^*^P < 0.05.

Based on two key observations from mice on folate-deficiency diet in this study, dysplasia of mouse fetuses and hypomethylation of the GNAS imprinting cluster, especially in the Exon1A/GNAS gDMR in embryonic brain, we speculated that the expression of genes controlled by involving gDMRs might be altered. Indeed, the expression level of *Nespas*, which is controlled by the Nespas gDMR, increased significantly in fetal brains derived from the MD and PD groups (*P* < 0.05; Figure 5A). In addition, the expression levels of *Exon1A* and *GNAS*, which are under the control of the Exon1A/GNAS gDMR, were also significantly elevated in fetal brains in the MD and PD groups (*P* < 0.05; Figure 5A). Similar comparison were evaluated in 10 paired brain tissues of human NTD cases and controls, as shown in Figure 5B, *Nespas* gene expression level showed a greater than 1.8-fold increase in NTDs, compared with that in controls (3.90 ± 1.09 in NTDs vs. 2.19 ± 1.21 in controls, *p =* 0.003), whereas no difference was detected in *Gnas* gene expression level between NTDs and controls.

Since we observed disastrous consequences in pregnancy outcome from folate deficiency mice and effect of folate deficiency on GNAS imprinting, and taking into account the fact that cyclic AMP signaling is known to be regulated by the GNAS gene cluster, we then measured cAMP levels in fetal brain tissues from folate-deficient groups and controls. As shown in Figure 5C, cAMP levels increased in all folate-deficient groups, whereas a profound elevation in cAMP levels was seen in the PD and in BPD groups. Furthermore, after segregating MD group based on resorption of fetuses, we observed a three folds increase in cAMP signaling in the MD group with resorption (Figure 5D).

Taken together, data from this study suggest that folate deficiency in either parent may result in a disturbed GNAS imprinting before fertilization and continue through embryogenesis, leading to abnormal fetus development possibly through altering cAMP signaling.

## DISCUSSION

It has been well accepted that folic acid deficiency during pregnancy increases the risk of NTDs, and changes in gene methylation regulation during embryogenesis are believed to be involved in the process. In this study, we presented data demonstrating that GNAS imprinting was affected by both maternal and paternal folic acid deficiency. Aberrant GNAS imprinting induced by a folate-deficient diet occurred prior to gametogenesis. One of the three DMRs, Exon1A/GNAS gDMR, lost its imprinting in both spermatozoa and oocytes from mice fed with a parental folate-deficient diet. Loss of imprinting in Exon1A/GNAS gDMR was maintained to fetuses starting from gametes. Aberrant methylation in the GNAS imprinting cluster altered the chromatin structure and disturbed gene functions through abnormal regulation of gene expression. Finally, we presented evidence that alteration in cAMP signaling might be the cause of multi-system disorders in embryogenesis under folic acid deficiency conditions. Our data provide experimental evidence supporting that aberrant GNAS imprinting is an attribute to NTDs in folic acid metabolism regulation during embryogenesis.

GNAS is a complex imprinted gene cluster controlled by multiple promoters [36]. Until now, three DMRs including two candidate imprinting control regions (ICRs) have been found in the GNAS cluster: GNAS gDMR, Nespas gDMR and paternal Nesp DMR [38]. In human NTD cases, we found that there was a significant loss of imprinting in Nespas gDMR, which belongs to the principal ICR of GNAS cluster [37]. As to imprinting regulation, gDMRs in ICRs are important not only because they are establish during gametogenesis and inherited by offspring, but also because they are hallmarks indicative of the establishment of imprinting in other regions of the whole imprinted gene cluster [38]. We further validated role of folic acid deficiency in pregnancy and its effects on GNAS imprinting in offspring in mouse model and our data clearly show that folic acid deficiency could directly reduce the methylation level in GNAS imprinted region. This supports the hypothesis that periconceptional folate deficiency increased the ratio of birth defects involving the impairment of imprinting establishment in the GNAS cluster.

Epigenetic marks established during gametogenesis are dynamic and flexible as there are critical windows in embryogenesis during which these epigenetic marks can be modified by nutritional factors [39]. Two major periods of epigenomic programming occur in mammals during embryogenesis: the first starts in gametogenesis, and ends with the establishment of germ cell-specific epigenetic profile; the second phase occurs in preimplantation embryos, during which epigenome is reprogrammed with the exception of imprinted genes and repeat sequences [39, 40]. Given that gDMRs of imprinted genes escape from the second wave of epigenomic programming, proper imprinting establishment in the first phase then becomes essential for normal embryogenesis. Any epigenetic defects in these imprinting regions induced by malnutrition during gametogenesis will be involved in the epigenetic inheritance of diseases. In this study, we demonstrated that parental folic acid deficiency altered GNAS imprinting in oocytes and spermatozoa, especially in the Exon1A/GNAS germline DMR. Such imprinting defects were passed on to the offspring, and led to abnormal expression of genes involved in development and controlled by these DMRs. These observations indicate that preconception diet and overall health status may be of equal importance in mothers and fathers, as has been suggested by recent studies [41, 42], but they play different roles in the development of the embryo. At this point, we can only speculate that imprinting defects in the Nespas and Nesp DMRs might be closely connected with the impaired status of Exon1A/GNAS DMR imprinting induced by a maternal folic acid-deficient diet during pregnancy. However, once Exon1A/GNAS DMR imprinting disturbed during gametogenesis, the defaults will extend throughout embryogenesis. In this folic acid-deficient diet model, loss of imprinting on Exon1A/GNAS DMR was seen as early as during oogenesis, and remained to fetal brain tissues at E13.5 and E18.5. Importantly, these results indicate that there are environmentally sensitive regions in the GNAS imprinting cluster that respond to diet during gametogenesis and regulate a so-called “imprinting landscape” that influences the whole imprinting cluster and subsequent disease in the offspring.

It has been well known that folate deficiency in pregnancy causes a variety of birth defects [22]. Furthermore, it has been shown that adequate folate intake is important during the reproductive period not only for women but also for men as well, and birth defects in offspring caused by disturbed maternal and paternal folate status have been investigated and adverse effects were observed [16]. By comparing pregnancy outcomes among different folic acid deficient animal groups, we presented direct evidence that defects in offspring could be the consequence of either maternal or paternal folate deficiency, or both. Interestingly, differentiated reproductive outcomes were observed between the maternal and paternal folic acid deficiency animal groups. Maternal folate deficiency predominantly affected the number of offspring with an increase in resorption, while paternal folate deficiency had a profound effect on the size of offspring, such as the crown–rump length. Such findings that paternal folate deficiency limited growth while maternal folate deficiency decreased live birth rates, are consistent with the expected roles of imprinting genes, in that paternal gene imprinting tends to be growth limiting (enhancement) while maternal imprinting is often aimed to conserve resources for her own and current and subsequent offspring [43].

The imprinted GNAS gene cluster has been reported to play important roles in controlling fetal growth [31, 32] and cell proliferation, and participates in IUGR [33–35, 44]. Therefore, we attempted to elucidate its direct effect on offspring birth defect by analyzing expression of genes under the control of GNAS which have been shown to be critical for normal embryonic development. Our results confirm that periconceptional folate deficiency alters the methylation patterns of the maternal gDMRs in the GNAS imprinting cluster and influences the expression of genes under the control of these gDMRs. Open chromatin architecture in the GNAS cluster might explain the regulation of gene expression by hypomethylation in which more sites in the chromatin are exposed to binding by transcription factors. Studies have suggested that the expression level of GNAS, which is part of the MAPK/ERK pathway and regulates cell proliferation through intracellular cyclic AMP, is increased in cases of IUGR [36, 45]. Intracellular cyclic AMP aberrations in the folic acid-deficient cells might be the linkage that explains how the regulation of folic acid in pregnancy regulates embryo development through gene imprinting. Moreover, since cyclic AMP is an important second messenger, possible aberrations in multi-pathways under controlled by cyclic AMP could be explanations for the multi-system dysontogenesis in NTDs.

In conclusion, our data suggest that folic acid levels in pregnancy regulate normal GNAS gene imprinting during embryogenesis. Although we cannot define the direct link between development defects and imprinting aberrancies under low folic acid conditions, our results indicate that the establishment of gene imprinting in the GNAS cluster is closely associated with folic acid metabolism during embryogenesis. This is a new perspective for explaining the molecular mechanisms by which folate supplementation in pregnancy provides protection from abnormal developmental features such as NTDs.

## MATERIALS AND METHODS

### NTD sample collection

Stillborn human fetuses with NTDs were collected in local county hospitals in Shanxi Province. China from March 2004 to July 2010. Pathological diagnosis of NTDs was performed according to the International Classification of Disease, Tenth Revision, Codes Q00.0, Q05.9, and Q01.9. The classification methods were based on those devised by Cabaret *et al.* in 2007 [36]. A routine prenatal checkup, questionnaire and an autopsy report were completed for all couples donating fetuses with NTDs and controls. None of the donor mothers had taken pre-conception folic acid supplementation. Pregnancies in which the mothers had been exposed to anti-folic acid or known anti-methylation medications, or those displaying pathological malformations or IUGR, were excluded from the study. The study protocol was reviewed and approved by the Ethics Board of Capital Institute of Pediatrics (IRB00008963), and written informed consent was obtained from all participants. All samples were stored at −20°C in local hospitals before being shipped, on ice, to the study laboratories.

### Experimental animals and diets

Protocols for all animal experiments were approved by the Capital Institute of Pediatrics Ethics Committee (IRB00008963). Animals were housed in individually ventilated cages (IVC-II) under a 14 h light/10 h dark cycle in a temperature- and humidity-controlled room and received free access to food and water. Eight-week-old C57BL/6J mice were maintained on a normal diet until 12 weeks of age, when they were randomly divided into two groups according to diet: 1) a folate-supplemented group receiving a normal diet with 2 mg folate per kg diet; and 2) a folate-deficient group receiving a diet from which folate was excluded. The compositions of each diet are shown in Supplementary Table 1 [25, 46]. The mice were maintained on the experimental diets for 4 weeks. For mating, male and female mice were randomly divided into four groups for crossing: paternal folate-supplemented × maternal folate-supplemented (PS), paternal folate-deficient *×* maternal folate-supplemented (PD), paternal folate-supplemented *×* maternal folate-deficient (MD), paternal folate-deficient *×* maternal folate-deficient (BPD) groups. Mating was confirmed by the presence of a vaginal plug. The day on which a plug was observed was designated embryonic day (E) 0.5. Folate-deficient or folate-supplemented diets were maintained throughout breeding and gestation until the animals were euthanized on E 18.5. The body weights of the dams were measured once a week after they had been assigned to their diet groups. At E 18.5, blood was collected from the eyeball and dams were euthanized by cervical dislocation. Gravid uteri were removed, and all implants and resorption sites were recorded. Embryos were dissected free of the extra-embryonic membranes under a stereomicroscope, and embryos and placentas were weighed. A sample of each embryo was fixed in 10% neutral buffered formalin for histology. Brains and livers were removed from the embryos, flash-frozen in liquid nitrogen and stored at −80°C. Mice that did not mate were killed by exsanguination under ether anesthesia and gametes were collected.

### Collection of oocytes and spermatozoa

Mice were euthanized by cervical dislocation. Both caudal epididymides were removed aseptically from the male mice and placed into 500 μL TYH buffer in a 35 mm culture dish (Corning, NY, USA). The epididymides were minced 5–6 times using forceps and scissors, and spermatozoa were allowed to disperse by gently shaking the dish by hand for 3–5 min at room temperature. Ovaries were collected from female mice and minced in TYH buffer; dispersed oocytes were collected using micropipettes.

### Cell culture

Mouse embryonic stem cells (ESCs; Sv/129) were obtained from Xuanwu Hospital (Beijing, China), and maintained as mitotically inactivated primary mouse embryonic fibroblasts prior to culture under feeder-free conditions. ESCs were seeded onto culture dishes coated with 0.2% gelatin (Sigma-Aldrich, St Louis, MO, USA) and maintained in folate-free Dulbecco’s modified Eagle’s medium (Sigma-Aldrich, St Louis, MO, USA) supplemented with 0.1 mMb-mercaptoethanol, nonessential amino acids, 2 mM glutamate, 15% fetal bovine serum (all purchased from Invitrogen Life Sciences, Carlsbad, CA, USA), 4 mg/l folate (Sigma-Aldrich, St Louis, MO, USA) and 1000 U/ml leukemia inhibitory factor (Millipore, Billerica, MA, USA). Cells were passaged every 3 days. After three passages under folate-normal (4 mg/l folate) conditions, mouse ESCs were divided into two groups; cultured in folate-normal dedium (FN) or medium without folate (folate-free group, defined as FF). Mouse embryonic bodies (EBs) were differentiated from ESCs after removal of leukemia inhibitory factor [47]. Cells were maintained at 37°C in a humidified atmosphere with 5% CO_2_. Medium changes were performed daily.

### Folate concentration assays

Folate concentrations in maternal serum were determined using a high-performance liquid chromatography tandem mass spectrometry system (HPLC-MS/MS). Folate contents of mouse maternal peripheral blood, mouse placentas and brains from human fetuses were measured using a competitive receptor-binding immunoassay (Chemiluminescent Immunoenzyme Assay Access Immunoassay system II, Beckman Coulter, Krefeld, Germany) according to the manufacturer’s instructions.

### DNA methylation assay

#### DNA extraction

Genomic DNA was extracted from embryonic brains, embryonic liver, placentas, maternal liver, EB cells of mouse origin and brains from human fetuses with NTD using the Maxwell^®^ 16 Tissue DNA Purification kits (Promega, Madison, WI, USA) according to the manufacturer’s instructions.

#### Bisulfite treatment

Aliquots of 500 ng DNA from each sample were bisulfite-converted using Methylamp DNA Modification kits (Epigentek, Farmingdale, NY, USA) according to the manufacturer’s instructions.

### Methylation analyses

Three DMRs have been identified in the GNAS cluster: an extensive germline maternally methylated region at the Nespas promoter; a maternally methylated germline region at the Exon1A promoter; and a paternally methylated region spanning the Nesp promoter. DNA methylation at the indicated loci was measured using the Sequenom MassARRAY platform (CapitalBio, Beijing, China). This system uses matrix-assisted laser desorption/ionization time-of-flight (MALDI-TOF) mass spectrometry in combination with RNA base-specific cleavage (MassCLEAVE). A detectable pattern was then analyzed for its methylation status. Polymerase chain reaction (PCR) primers were designed using Methprimer (http://epidesigner.com). The PCR primers specific for bisulfate-converted DNA for the DMRs in the GNAS imprinting cluster are listed in Supplementary Table 2. The locations of the amplicons used for methylation analysis are shown by the short bars under each DMR (Figure 2). The methylation ratios of the spectra were generated using Epityper software version 1.0 (Sequenom, San Diego, CA, USA).

### EpiQ assay

ESC cells were grown and differentiated to EBs in 48-well plates and treated with EpiQ Chromatin Analysis kits (BioRad, Hercules, CA, USA). In this assay, heterochromatin is inaccessible to the nuclease, rendering it protected from digestion and available for subsequent quantitative (q)PCR. Cycle delays (ΔCq) with and without nuclease digestion were used to evaluate chromatin accessibility within the GNSA gDMR region. Primers for the GNSA gDMR assay are listed in Supplementary Table 3. Two other genes, constitutively expressed *GAPDH* and the epigenetically silenced *RHO* (according to the manual of the EpiQ Chromatin Analysis kit) were also included as control genes to confirm the optimal assay conditions. All qPCR runs were carried out on a ABI 7500 Fast Thermal cycler (Applied Biosystems, Foster City, CA, USA) using the EpiQ^™^ chromatin SYBR^®^ Green supermix (BioRad, Hercules, CA, USA) after genomic DNA extraction.

### Gene expression analysis

Total RNA was extracted using RNeasy® Mini kits (QIAGEN, Hilden, Germany). For reverse transcription PCR, 1 μg of total RNA was reverse-transcribed into cDNA using the Protoscript^®^ First Strand cDNA Synthesis kits (NEB, Beverly, MA, USA) according to the manufacturer’s instructions. Real-time qPCR was performed using a 7500 Fast Real-Time PCR system (Applied Biosystems, Foster City, CA, USA). Samples for qPCR were run in triplicate. Relative mRNA levels were compared using the 2^−DDCT^ method, with *GAPDH* as a control. For primer sequences see Supplementary Table 2.

### NanoString nCounter expression assay

NanoString nCounter Analysis System (NanoString Technologies, Seattle, WA, USA) was used to evaluate gene expression in human cases. This system measures the relative abundance of each mRNA transcript via a multiplexed hybridization assay and digital readouts of fluorescent probes. We used an nCounter CodeSet (NanoString Technologies) containing biotinylated capture probes for the *GNAS* and *Nespas* genes and five housekeeping genes and reporter probes attached to color barcode tags, according to the nCounter™ code-set design. These were hybridized in solution to 100 ng of total RNA of fetus brain tissue for 18 h at 65°C according to the manufacturer’s instructions.

Hybridized samples were loaded into the nCounter Prep Station for post-hybridization processing. Hybridized samples were purified and immobilized on the deck of the Prep Station in a sample cartridge for data collection, and target mRNA was quantified in each sample using the nCounter™ Digital Analyzer. Quantified expression data were analyzed using NanoString nSolver Analysis Software.

After performing image quality control using a predefined cut off value, we excluded outlier samples using a normalization factor based on the sum of positive control counts greater than threefold. The counts of the probes were then normalized using the geometric mean of the five housekeeping genes and log2 transformed for further analysis.

### Cyclic AMP assay

The cAMP concentrations in mouse fetal brain tissues were measured using mouse cAMP enzyme-linked immunosorbent (ELISA) kits (FK-QZ3275, China).

### Statistical analyses

Data were analyzed using the SPSS-17.0 software package (McGraw-Hill Inc., New York, NY, USA). All *P* values were two-sided, and *P* < 0.05 was considered to be significant. Independent Student’s *t-*tests were performed to evaluate the significance of any difference between groups. A χ^2^ test was used to evaluate differences between groups. Bivariate correlation analysis was performed using Pearson’s correlation coefficient (*r*).

## SUPPLEMENTARY MATERIALS FIGURES AND TABLES


