# Supplementary material for: Altered GNAS imprinting due to folic acid deficiency contributes to poor embryo development and may lead to neural tube defects
Source: Oncotarget. 2017 Nov 28;8(67):110797–810. doi: 10.18632/oncotarget.22731 (PMC5762285; doi:10.18632/oncotarget.22731)
Supplement: Supplementary file 1 [file oncotarget-08-110797-s001.pdf]

Altered GNAS imprinting due to folic acid deficiency contributes to poor embryo development and may lead to neural tube defects

SUPPLEMENTARY MATERIALS

Tissue specific methylation levels of DMRs in the Gnas imprinting cluster in E13.5 mouse fetuses in maternal folate-deficient (MD) group

The effect of maternal folate deficiency on Gnas imprinting patterns was measured in various tissues. Fetal liver, maternal liver and placental tissue, which are linked to folate reserves and exchange between dam and offspring were selected. Data are shown in Supplementary Table 3. In fetal liver, there was a small but significant methylation decrease in the Exon1A/Gnas gDMR in the MD group (33.5% *vs.* 35.2%). However, in the placenta methylation level in this region was increased (25.4% *vs*. 23.2%; $P < 0.05$). In the case of Nespas gDMR, no significant difference in the overall methylation level of this region was found in fetal liver and placental tissue, similar results were found in the Nesp DMR region, which is paternally imprinted. In the maternal liver, although the folate concentration was significantly lower in the MD group, the methylation levels of all three DMRs, whose methylation patterns had already been established, were not affected by periconceptional folate deficiency. The greatest difference in the methylation level of the Exon1A/Gnas gDMR between the MD and PS groups was found in the embryonic brains. Meanwhile, our data show a striking reduction in the methylation level at chr2:174,120,698–174,120,842, located at the 3′ region of the Nespas gDMR in fetal brain (33.1% *vs*. 46.1%; $P < 0.001$, Supplementary Figure 2).

Supplementary Table 1: Basic composition of mouse diet (g/kg)

Ingredient	Control	Folate-deficient diet group
Cornstarch	380.500	380.500
Amino acids mix*	200.000	200.000
Dextrinized cornstarch	152.000	132.000
Sucrose	100.000	100.000
Soybean oil	70.000	70.000
Fiber	50.000	50.000
Mineral mix*	35.000	35.000
Vitamin Mix*	10.000	–
w/o Folate Vitamin Mix*	–	10.000
Choline bitartrate	2.500	2.500

Supplementary Table 2: List of primers used in manuscript

Primer	Left Primer	Right Primer	Region	Target Location
**mouse**				
Nespas 1	5′-aggaagagagTATAGTAAGATGTTTTG ATTGAATAGAAGG-3′	5′-cagtaatacgactcactatagggagaaggctTCTAAAA ACTTAATTCAAATCCTAATCT-3′	Nespas DMR	chr2:174,119,977–174,120,276
Nespas 2	5′-aggaagagagTTGAATTTTGTATAAGG TGAAAGGT-3′	5′-cagtaatacgactcactatagggagaaggctAAAAACC TTCTCTTTCCATTTTCAT-3′	Nespas DMR	chr2:174,120,331–174,120,629
Nespas 3	5′-aggaagagagAGGTGGGAAGGTTAG GGATTTTTAT-3′	5′-cagtaatacgactcactatagggagaaggctCTCTCTT TCTCCATAAACCTAACCC-3′	Nespas DMR	chr2:174,120,698–174,120,842
Nespas 4	5′-aggaagagagTAGGTTAGTTATTGGGT TGGGGTTA-3′	5′-cagtaatacgactcactatagggagaaggctCCCAAAA AATAATCCATAAAAATCC-3′	Nespas DMR	chr2:174,120.803–174,121,095
Nespas 5	5′-aggaagagagGGTTAATTGGGTTTTAA TTATTAGTTT-3′	5′-cagtaatacgactcactatagggagaaggctAACTTAA CCCCCAAAACACTCAC-3′	Nespas DMR	chr2:174,120,869–174,121,143
Nespas 6	5′-aggaagagagGGGTATATTGTAAGATG TATGTTGTGTGT-3′	5′-cagtaatacgactcactatagggagaaggctCCAAACT TTAAACCAAAATTCAAAA-3′	Nespas DMR	chr2:174,121,216–174,121,469
Exon1A/ Gnas 1	aggaagagagAGTTTTTTTAGGTGGGA GAGGTG	cagtaatacgactcactatagggagaaggctTTAAAACAA TTCTCCAAAAAATTCA	Exon1A/Gnas DMR	chr2:174,152,547–174,152,657
Exon1A/ Gnas 2	5′-aggaagagagTTATTTGGGAAGTTGA GGTAAGAA-3′	5′-cagtaatacgactcactatagggagaaggctCCTAAAC CTACCTCCCAATTTTC-3′	Exon1A/Gnas DMR	chr2:174,152,875–174,152,992
Exon1A/ Gnas 3	5′-aggaagagagAAAATTGGGAGGTAGG TTTAGGAG-3′	5′-cagtaatacgactcactatagggagaaggctCCCCAAC CTCTTCAAAAAAC-3′	Exon1A/Gnas DMR	chr2:174,152,971–174,153,211
Exon1A/ Gnas 4	5′-aggaagagagTTTTGTTGTTGTTGGG TGTTTTT-3′	5′-cagtaatacgactcactatagggagaaggctATAACCA TCTTCAACATAATACCCC-3′	Exon1A/Gnas DMR	chr2:174,153,775–174,154,070
Exon1A/ Gnas 5	5′-aggaagagagAGATGGAGAAGAAGTT GTTGGAGTA-3′	5′-cagtaatacgactcactatagggagaaggctTCTTCAT CAATATCACTATCCCATTC-3′	Exon1A/Gnas DMR	chr2:174,154,289–174,154,556
Nesp 1	aggaagagagGAGGAGAGTAATGGAGT AGGAGAGTT	cagtaatacgactcactatagggagaaggctAATTAAAAT CTTCAAACCCCAACTT	Nesp DMR	chr2:174,110,043–174,110,300
Nesp 2	aggaagagagTTGAAGATTAATTTTTGT AGGAGGG	cagtaatacgactcactatagggagaaggctAACTCTTCA AATCTCTCCAAAACAC	Nesp DMR	chr2:174,111,108–174,111,358
human				
Nespas 1	5′-aggaagagagTTTTTAAGGTTAAGAA GGTATTTTTGG-3′	5′-cagtaatacgactcactatagggagaaggctCTTTAAC TAAACCCCCTCCTCC-3′	Nespas DMR	chr20:56,863,612–56,863,900
Nespas 2	5′-aggaagagagTTGTTAGAAAGTTTTA GGGAGGGAT-3′	5′-cagtaatacgactcactatagggagaaggctCCACCAA CCTAACCAAAAAAATC-3′	Nespas DMR	chr20:56,863,912–56,864,157
Exon1A/ Gnas 1	aggaagagagTGTTTTTTGGTTTGTTTT TGTTTTT	cagtaatacgactcactatagggagaaggctCATAACCAT CTTCAACATAATAACCC	Exon1A/Gnas DMR	chr20:56,898,139–56,898,354
Nesp 1	5′-aggaagagagGTTAAATTGGGGAGTT TGAGGG-3′	5′-cagtaatacgactcactatagggagaaggctCAACAAA CCTTTAACCACCAAAAC-3′	Nesp DMR	chr20:56,849,351–56,849,547
Nesp 2	5′-aggaagagagTTTTAAATAAGTTGGTT TTTTTAGGTGT-3′	5′-cagtaatacgactcactatagggagaaggctCTTAATTA TCAACTCTAAAACCTAAACTCC-3′	Nesp DMR	chr20:56,849,718–56,849,950
Real-Time PCR				
Nespas	GATGCCCTAAATCGCCAAGG	TGTGCAGGGTTCAGCATCTC		
Exon1A	ACATAGCTAACCCAAGGAGCAC	CCTGCACTTTAGTGGCCTTCT		
Gnas	TCGGCAACAGTAAGACCGAG	TGCTTTTGCCAGACTCTCCA		
Gapdh	TGTGTCCGTCGTGGATCTGA	CCTGCTTCACCACCTTCTTGA		
EpiQ				
GNAS	GGCGTGCCCGTACCCCTCAATAGAT			
GNAS	CTAACCAGCCTGATGACGCCCCAAC			

Supplementary Table 3: Tissue specific methylation levels of DMRs in Gnas imprinting cluster between maternal folate deficient group and the normal group[1]

		Nespas DMR (%)	Exon1A/Gnas DMR (%)	Nesp DMR (%)
**Fetal brain**	**MD**	43.5 ± 2.6	33.4 ± 2.8*	41.1 ± 3.1
	**PS**	42.7 ± 2.2	38.3 ± 3.0	41.4 ± 4.7
**Fetal liver**	**MD**	37.6 ± 2.1	33.5 ± 0.9*	42.7 ± 2.9
	**PS**	38.9 ± 1.6	35.2 ± 1.3	41.0 ± 2.1
**placenta**	**MD**	40.4 ± 1.7	25.4 ± 2.0*	42.9 ± 2.0
	**PS**	39.4 ± 1.2	23.2 ± 1.0	44.6 ± 2.6
**Maternal liver**	**MD**	44.1 ± 4.2	39.4 ± 3.7	47.0 ± 8.0
	**PS**	46.8 ± 5.9	41.3 ± 4.7	46.9 ± 6.9

All values are the mean ± SD. *$P \leq 0.05$ by Student's *t*-test.

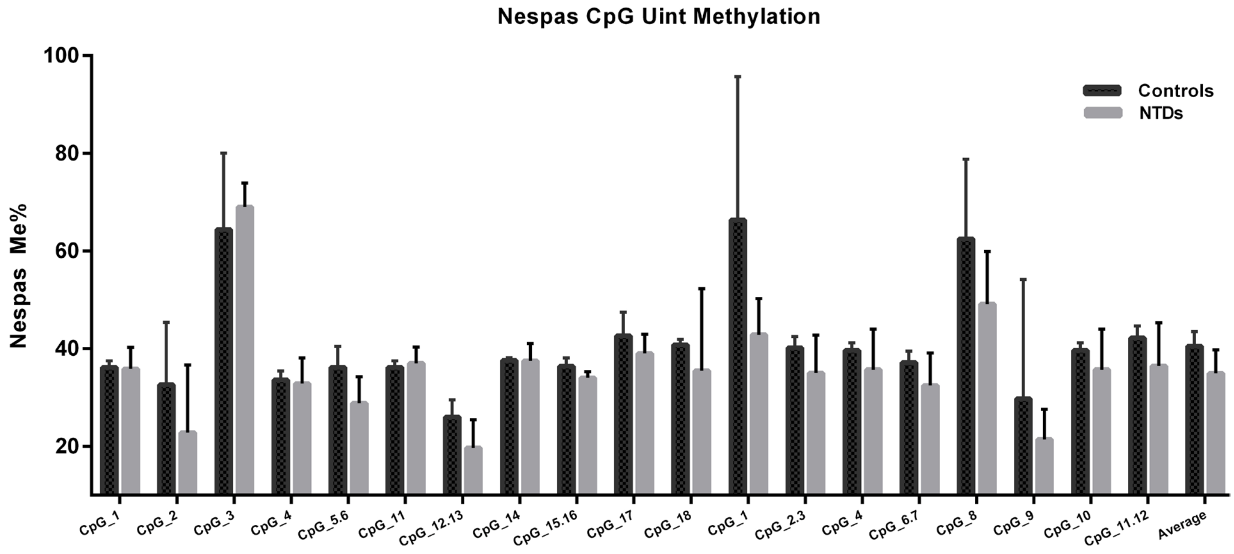

Supplementary Figure 1: Methylation level of CpG sites in the Nespas gDMR in brain tissues between NTD cases and controls. All values are the mean ± SD, *$P < 0.05$.

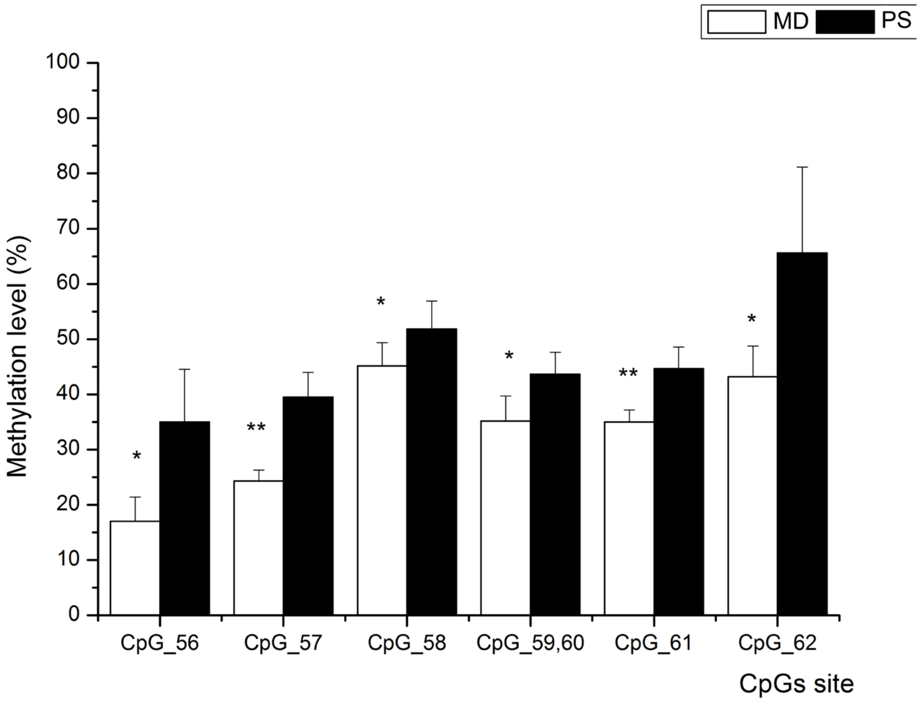

Supplementary Figure 2: The methylation levels of every CpG site at chr2:174,120,698–174,120,842 located in 3′of the Nespas gDMR in E 13.5 fetal mouse fetal brain. All values are mean ± SD, $^{*}P < 0.05$.
